# BOMET-QoL-10 questionnaire for breast cancer patients with bone metastasis: the prospective MABOMET GEICAM study

**DOI:** 10.1186/s41687-019-0161-y

**Published:** 2019-12-21

**Authors:** A. Barnadas, M. Muñoz, M. Margelí, J. I. Chacón, J. Cassinello, S. Antolin, E. Adrover, M. Ramos, E. Carrasco, M. A. Jimeno, B. Ojeda, X. González, S. González, M. Constenla, J. Florián, A. Miguel, A. Llombart, A. Lluch, M. Ruiz-Borrego, R. Colomer, S. Del Barco, On behalf of GEICAM, Spanish Breast Cancer Group

**Affiliations:** 1 Medical Oncology Department, Hospital de la Santa Creu I Sant Pau, C/Sant Antoni Maria Claret, 167, 08041 Barcelona, Spain; 2 Centro de Investigación Biomédica en Red de Oncología, CIBERONC-ISCIII, Madrid, Spain; 3 Medical Oncology Department, Hospital Clinic i Provincial, C/ Villarroel n° 170, 08036, Barcelona, Spain; 4 Medical Oncology Department, Ctra, Hospital Germans Trias i Pujol, Canyet s/n, 08916 Badalona, Barcelona, Spain; 5 Medical Oncology Department, Hospital Virgen de la Salud, Avda. Barber, n° 30, 45005 Toledo, Spain; 6 Medical Oncology Department, Hospital General de Guadalajara, C/ Donantes de Sangre, s/n, 19002 Guadalajara, Spain; 7 Medical Oncology Department, Complejo Hospitalario U. A Coruña, C/ Xubias de Abaixo s/n, 15006 A Coruña, Spain; 8 Medical Oncology Department, Complejo Hospitalario Universitario de Albacete, C/ Hermanos Falcó n° 37, 02006 Albacete, Spain; 9 Medical Oncology Department, Centro Oncológico de Galicia, C/ Doctor Camilo Veiras s/n, 15009 A Coruña, Spain; 10 GEICAM (Spanish Breast Cancer Group), Avda. de los Pirineos n° 7, 28703 San Sebastián de los Reyes, Madrid, Spain; 11 Medical Oncology Department, Hospital General de Catalunya, Carrer de Pedro Pons 1, 08195 Sant Cugat del Valles, Barcelona Spain; 12 Medical Oncology Department, Hospital Mutua de Terrassa, Barcelona, Plaza del Dr. Robert n°5, 08221 Terrassa, Barcelona Spain; 13 Medical Oncology Department, Complejo Hospitalario De Pontevedra, Calle Mourente s/n, 36071 Pontevedra, Galicia Spain; 14 Medical Oncology Department, Hospital Comarcal de Barbastro, Ctra. Nacional 240, s/n, 22300 Barbastro, Huesca Spain; 15 Medical Oncology Department, Hospital Althaia Manresa, C/ Dr. Joan Soler, s/n, 08243 Manresa, Barcelona Spain; 16 Medical Oncology Department, Hospital Arnau de Vilanova, Avda. Alcalde Rovira Roure, 80, 25198 Lleida, Spain; 17 Hospital Clínico Universitario de Valencia, Biomedical Research Institute INCLIVA, Valencia, Spain; 18 Medical Oncology Department, Hospital Virgen del Rocío, Avda. Manuel Siurot, s/n, 41013 Sevilla, Spain; 19 Medical Oncology Department, Hospital Universitario La Princesa, C/ Diego de León n° 62, 28006 Madrid, Spain; 20 Medical Oncology Department, Hospital U. Josep Trueta, Avda. De França s/n, 17007 Gerona, Spain

**Keywords:** Metastatic breast cancer, Bone metastasis, Quality of life, Psychometric, Treatment, Assessments

## Abstract

**Background:**

Bone metastasis (BM) is the most common site of disease in metastatic breast cancer (MBC) patients. BM impacts health-related quality of life (HRQoL). We tested prospectively the psychometric properties of the Bone Metastasis Quality of Life (BOMET-QoL-10) measure on MBC patients with BM.

**Methods:**

Patients completed the BOMET-QoL-10 questionnaire, the Visual Analogue Scale (VAS) for pain, and a self-perceived health status item at baseline and at follow-up visits. We performed psychometric tests and calculated the effect size of specific BM treatment on patients´ HRQoL.

**Results:**

Almost 70% of the 172 patients reported symptoms, 23.3% experienced irruptive pain, and over half were receiving chemotherapy. BOMET-QoL-10 proved to be a quick assessment tool performing well in readability and completion time (about 10 min) with 0–1.2% of missing/invalid data. Although BOMET-QoL-10 scores remained fairly stable during study visits, differences were observed for patient subgroups (e.g., with or without skeletal-related events or adverse effects). Scores were significantly correlated with physician-reported patient status, patient-reported pain, symptoms, and perceived health status. BOMET-QoL-10 scores also varied prospectively according to changes in pain intensity.

**Conclusions:**

BOMET-QoL-10 performed well as a brief, easy-to-administer, useful, and sensitive HRQoL measure for potential use for clinical practice with MBC patients.

**Trial registration:**

NCT03847220. Retrospectively registered on clinicaltrials.gov (February the 20th 2019).

## Introduction

Breast cancer represents 25% of all cancer cases worldwide [1]. Between 6% and 10% of them will present as metastatic breast cancer (MBC) at diagnosis [2]. Between 30% and 40% of those receiving adjuvant or neoadjuvant chemotherapy for early-stage disease will eventually develop MBC [3, 4]. Thanks to the incorporation of new therapeutic agents to cancer treatment and a better disease management, the median survival has increased in patients with advanced disease over the years. Patients live longer and health-related quality of life (HRQoL) becomes a great outcome of interest in MBC-related research [3, 4].

Bone metastasis (BM), the most common site of disease in MBC patients [5], greatly impacts HRQoL due to its high morbidity [6–9]. In addition, treatment-related toxicities are generally under-reported by physicians when compared to patients´ reports [10]. Surprisingly, how and to what extent MBC symptoms, MBC-related treatments, or palliative care may affect patients´ HRQoL remains understudied [11–14].

Better understanding of the disease’s overall impact on HRQoL requires questionnaires designed to assess patients’ perception of their pain and other potentially life-limiting symptoms. The questionnaire designed by the European Organization for Research and Treatment of Cancer (EORTC), QLQ-C30, and the functional assessment of cancer therapy (FACT-G) assess general cancer-related HRQoL issues. The corresponding breast cancer modules focus on image and sexual life issues, lymphedema of the arm, and surgery incision pain, i.e., challenges found in both metastatic and non-metastatic patients. However, 50–70% of patients with BM present with bone pain and related threats to HRQoL [15]. The Quality of Life Questionnaire-Bone Metastasis-22 (QLQ-BM22), a 22-item submodule of QLQ-C30 [15], and Bone Metastasis Quality of Life (BOMET-QoL), a 25-item questionnaire [16], were designed to fill this gap by targeting cancer patients with BM.

BOMET-QoL was later reduced to 10 items (BOMET-QoL-10) and validated using a similar population of patients with BM. In that validation study, factorial analyses identified a single dimension which accounted for 45.8% of explained variance. The disease’s impact on HRQoL did not vary significantly across clinical variables such as number and location of bone metastases and time since diagnosis. However, it did vary significantly by presence, number, and duration of irruptive pain crises, pain management index (PMI), and Eastern Cooperative Oncology Group Performance Status (ECOG PS) [17]. Reported impact on HRQoL was lower with fewer irruptive pain crises when pain control was satisfactory (PMI = 0) or when ECOG PS score was between 0 and 1 [18].

In most cases, BOMET-QoL-10 questionnaire scores correlated statistically significantly, and at least moderately, with EORTC QLQ-30 and BOMET-QoL dimensions, confirming convergent validity. Also, BOMET-QoL-10 scores experienced a statistically significant increase (i.e., improved HRQoL) throughout the study [18]. The effect size between the baseline visit and the 6-month follow-up was 0.841. BOMET-QoL-10 showed the capacity to detect changes in perceived health status (between 3 and 6 months), pain perception, and ECOG PS score throughout the study. This indicates that BOMET-QoL-10 is able to detect changes in relevant conditions. The instrument also exhibits good internal consistency (Cronbach’s α =0.93) and good test-retest reliability (Intraclass Correlation Coefficient = 0.94). The result is a useful 10-item measure of HRQoL easy-to-use in both clinical trial interventions and in regular clinical practice [18]. Previously, Adrover and colleagues (2005) were unable to evaluate the psychometric properties and clinical utility for specific types of cancer, such as MBC, due to sample size limitations [16].

MBC patients with BM have disease-specific complications with particular impacts on HRQoL. Thus, the main objective of the present study was to evaluate the aplicability of BOMET-QoL-10 in this patient population, regardless of treatment received. Secondary objectives included: 1) Evaluating the differences on BOMET-QoL-10 scoring when patients have skeletal-related events (SREs) and BM symptoms; 2) Exploring the association between BOMET-QoL-10 scores and other measures of self-perceived health status and pain such as ECOG PS and Visual Analog Scale (VAS); and 3) Assessing whether the magnitude of the effect of BM treatment on patients´ HRQoL reaches clinical significance.

## Materials and methods

This was a prospective, observational, multicenter study of MBC patients who received systemic therapy according to local guidelines. MBC patients were eligible for inclusion with age above 18 and diagnosed with BM, ability to understand and complete the questionnaires, estimated life expectancy of at least 8 months, and absence of co-morbidities.

Study visits were pre-specifed in the protocol as a first baseline visit and 6 follow up visits at 4, 8, 12, 16, 20 and 24 months after baseline visit.

**Fig. 1:**
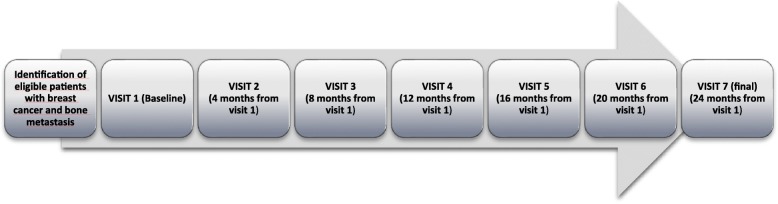
Study timeline

Figure 1 shows the study timeline. The following data were collected: 1) Basic socio-demographic data: age at study entry and family support; 2) Clinical data: date of diagnosis, stage of disease at diagnosis, site(s) and date of diagnosis of BM and visceral metastases at baseline; SREs, MBC-related symptoms, type of irruptive pain crises if any, treatments for breast cancer and for BM, chronic osteoarticular comorbidities and their treatment, other chronic diseases, and ECOG PS score were collected by the physician at baseline and throughout the study; 3) Safety measures: clinically-relevant adverse events (AE) and their grades and blood tests (especially creatinine, calcium, and albumin) were collected during each follow-up visit.

To assess HRQoL and perceived health data participants completed the BOMET-QoL-10 questionnaire (Additional file 1) and the VAS for pain at study visits in the hospital. The questionnaire was administered to the patients on paper by trained study site personnel. In the BOMET-QoL-10 patients were asked to respond to questions based on a 5-point Likert scale ranging from “never” (4) to “always” (0). The VAS for pain was a continuous 10 cm line where the patients have to mark the pain they were experiencing at that moment (left side of the line was no pain and the other end was unbearable pain). Perception of general health status was collected at baseline and reports of *changes* in perceived health status (compared to that at baseline) were collected at follow-up visits. General health status was based on both patient’s and health professional’s responses ranging from “very good” to “very bad” on a 7-point Likert scale.

### Statistical analysis

Initially, we calculated the need to recruit 241 patients based on the following assumptions: a standard deviation (SD) of 23 points for BOMET-QoL-10 scores (same as BOMET-QoL), a 20% patient drop-out rate, an estimation of HRQoL with a precision of ±3.25, and having at least 80% power to detect a difference ≥ 0.20 SD between BOMET-QoL-10 scores at baseline and at last follow-up (visit 7) with a 0.05 level of significance. However, due to recruitment limitations, we recalculated the target recruitment to 174 by slightly lowering our precision in estimating QoL to a ± 3.75 instead, while keeping the other parameters the same.

Categorical variables were described using frequencies and their differences were tested using Chi-square (*χ*^2^) or Fisher exact tests. Associations between ordinal variables were described using Kendall’s Tau. We used means, standard deviations, medians, and ranges (minimum and maximum) to describe continuous variables. Depending on variables’ distributions either Student t-tests or Mann–Whitney *U*-tests were used for comparisons. Likewise, we used Pearson r coefficients or Spearman’s rank correlation coefficients (ρ) to assess associations between variables of interest. Relationships between BOMET-QoL-10 scores and ECOG PS scores, perceived health status, and presence of SREs and AEs were analyzed.

To assess the BOMET-QoL-10 scores we calculated the mean score value in every visit for every item and a global standardized mean score of the whole questionnaire. This value was performed taking into account those questionnaires completely fulfilled (10 questions). The higher the BOMET-QoL-10, the higher quality of life (HRQoL).

To assess the clinical usefulness of our results we calculated the effect size of the questionnaire’s scores for patients receiving any specific medical intervention for BM, at any visit(s) during the study, versus patients who did not. The minimum effect size (d) for the difference between groups to be considered a clinically significant improvement in HRQoL was set at d ≥ 0.20. The effect size was calculated using the standardized mean difference, defined in this case as the ratio of the difference in mean scores between baseline and last follow-up and the standard deviation of scores at baseline.

To evaluate the correlation between the reduction in pain (according to pain VAS) and the HRQoL on the BOMET-QoL questionnaire, we compared the difference of mean values of those parameters between baseline and last follow-up visit.. We categorized this as: “Gets worse” if the difference was negative, “Stays the same” if the difference was zero and “Improves” if the difference was positive.

Finally, to evaluate the evolution of BOMET-QoL-10 scores, when explicitly mentioned, certain analyses only included those questionnaires with no missing data. All tests were two-tailed with an α = 0.05. Analyses were conducted using SPSS version 17.0 (SPSS Inc., Chicago IL, 2008).

## Results

From October 2007 to May 2010, 174 patients were recruited at 15 GEICAM hospitals distributed throughout Spain. One hundred seventy-two were eligible and enrolled in the study. Only 83 of them completed the last follow-up visit at 24 months. Missing data for specific questions or instruments varied across assessments, as reflected in in Tables 1 and 2.

**Table 1:** Baseline patient characteristics (*n* = 172)

Characteristics	Value
Age (years), median (Range)	58 (30–89)
Bone metastasis site, n (%)	
Spine	131 (76.2)
Ribs	78 (45.3)
Pelvis	92 (53.5)
Upper limbs	24 (14.0)
Lower limbs	48 (27.9)
Skull	37 (21.5)
Other	48 (27.9)
Visceral metastasis, n (%)	70 (40.7)
Patients with skeletal related events, n (%)	32 (18.6)
Pathologic fractures	8 (25.0)
Hypercalcemia	1 (3.1)
Medullar compression	1 (3.1)
Need for surgery	8 (25.0)
Need for radiation therapy	21 (65.6)
Patients with symptoms, n (%)	119 (69.2)
Lower back pain	44 (37.0)
Hip pain	32 (26.9)
Rib pain	27 (22.7)
Asthenia	39 (32.8)
Arthromyalgia	7 (5.9)
Other bone pain	8 (6.7)
Other	23 (19.3)
Patients with Irruptive Pain, n (%)	40 (23.3)
Incidental	29 (72.5)
Spontaneous	9 (22.5)
Other	3 (7.5)
Patients with non-osteoarticular comorbidities, n (%)	59 (34.3)
Chronic Obstuctive pulmonary disease (COPD)	1 (7)
Dislipemia	18 (30,5)
Cardiovascular disease	7 (11.9)
Arterial hypertension	35 (59.3)
Diabetes mellitus	11 (18.6)
Other	25 (42.4)

**Table 2:** Relationship between HRQoL according to BOMET-QoL-10 and pain VAS

Bomet-QoL-10 HRQoL questionnaire						
Visits	Pain VAS	Gets worse	No change	Improves	Correlation (Kendall’s Tau-b)	*p*-value
1 vs 2 *n* = 133	Gets worse	33	5	19	0.232	0.003
	No change	7	4	5		
	Improves	19	7	34		
2 vs 3 *n* = 106	Gets worse	37	6	13	0.466	< 0.0001
	No change	3	1	2		
	Improves	8	4	32		
3 vs 4 *n* = 97	Gets worse	23	6	9	0.271	0.002
	No change	5	1	7		
	Improves	15	5	26		
4 vs 5 *n* = 92	Gets worse	28	6	11	0.348	< 0.0001
	No change	5	1	2		
	Improves	12	0	27		
5 vs 6 *n* = 72	Gets worse	24	0	11	0.373	0.001
	No change	1	4	2		
	Improves	8	2	20		
6 vs 7 *n* = 69	Gets worse	25	3	8	0.432	< 0.0001
	No change	1	4	1		
	Improves	7	2	18		
1 vs 7 *n* = 79	Gets worse	31	0	12	0.464	< 0.0001
	No change	1	1	2		
	Improves	13	1	18		

*HRQoL* Health Related Quality of Life

Patients’ median age was 59 years (range: 30–89) and only 14% lived alone. The median time between breast cancer diagnosis and study enrollment was 4.4 years (Range: 0–36.8). Over half of the patients (52.7%) were stage IV at diagnosis. The most common BM sites were the spine (76.2%), pelvis (53.5%), and ribs (45.3%). In addition, visceral metastases were present in 40.7% of patients. SREs were reported in 18.6% of patients, the most common one being pathologic fractures (25.0%). Almost 70% of patients presented symptoms, mostly lower back pain (37.0%) and weakness (32.8%). Further, 23.3% of patients presented irruptive pain, of whom, 72.5% reported the pain to be incidental and clearly linked to oncological and neuropathic pain. Over 50% of patients presented chronic conditions, 19.2% were osteoarticular and 34.3% were non-osteoarticular comorbidities. (Table 1). More than half of the patients had a baseline ECOG PS score of 1 (51.2%) and 37.3% had a score of 0.

At baseline 54.7% of patients were receiving chemotherapy, 58,1% were on hormone therapy, and 12.2% received other treatments including bisphosphonates, palliative radiotherapy for bone metastasis or orthopedic surgery (Additional file 2). Patients could be receiving more than one treatment concurrently and they continued receiving treatments during follow-up visits. Overall, at each follow-up the proportion of patients receiving any one treatment grew smaller, except hormone therapy, but still with a marked downward trend. For instance, at the 24-month follow up, only 16.9% were receiving chemotherapy, 1.2% radiotherapy, 20.5% endocrine therapy, and 4.8% other treatments (analgesic, anti-inflamatory therapy and some rehabilitation). Patients were also treated for BM with bisphosphonates (79.1%) and pain relievers (37.8%) As with MBC treatment, the proportion of patients receiving treatment for BM tended to decrease at each follow-up (Additional file 3).

The vast majority of patients (80%) completed the BOMET-QoL-10 in less than 10 min in all visits, with a high completion rate. The small proportion of unanswered items throughout the entire follow-up ranged between 0% and 1.2%. Based on fully-completed questionnaires, we calculated the global average score for each visit from baseline (*n* = 172) to the 24-month follow-up (*n* = 83). There was no significant trend in BOMET-QoL-10 scores over the 7 visits. In fact, baseline and last follow-up global average scores (25.6 [SD 8.0] and 25.9 [SD 8.2], respectively) were were not statistically significant different (Additional file 4). Similarly, there were no a regular behavior for each of the 10 items of the questionnaire across visits and there were no items more likely to be skipped than others. However, there were two items with a tendency to receive lower scores, reflecting worse HRQoL, than the rest: “I feel tired” and “I have pain in some areas of my body such as my back, legs, hips, … that affect my life” and two items that tended to get higher scores reflecting better HRQoL, than other items: “I avoid family activities” and “I have an intense pain that doesn’t leave me alone.”

Further, as expected, BOMET-QoL-10 lower scores were negatively correlated with the number of BM-related symptoms (ρ = − 0.410, *p* < 0.001) (Additional file 5) as well as with VAS pain global standardized mean scores (ρ = − 0.542, *p* < 0.01). These patterns of correlations held true for the scores reported in every visit (Table 2). In addition, 32 patients whose pain improved between baseline and last follow-up visit (visit 1 vs 7, Table 2) reported significantly higher BOMET-QoL-10 average scores compared to those whose pain remained at the same level or worsened (29.4, SD = 7.7 vs.24.0, SD = 7.8; *p* = 0.003).

Most patients (85.5%) reported ECOG PS scores between 0 and 1. We compared BOMET-QoL-10 scores for each visit between two groups formed by patients with ECOG PS scores 0–1 versus those with ECOG PS scores > 1, to assess the differences in QoL within these groups. The former reported significantly higher BOMET-QoL-10 scores (range: 26.5 to 28.0) than the latter (range: 17.3 to 22.9). Differences between patients with ECOG PS 0–1 and > 1 were statistically significant overall and for every visit, except in visit 3 (Table 3). The BOMET-QoL-10 and perceived health status scores were also moderately inversely correlated for all 7 visits (range: − 0.6 to − 0.3; p < 0.001 for all).

**Table 3:** BOMET-QoL-10 scores throughout the study by ECOG PS

	Mean	SD	*p*
Visit 1 (*n* = 166)			
ECOG 0–1	26.5	7.6	0.003
ECOG > 1	19.8	9.2	
Visit 2 (*n* = 143)			
ECOG 0–1	26.5	7.9	0.002
ECOG > 1	18.0	8.6	
Visit 3 (*n* = 120)			
ECOG 0–1	26.7	7.4	0.111
ECOG > 1	22.9	7.2	
Visit 4 (*n* = 115)			
ECOG 0–1	27.2	7.6	0.000
ECOG > 1	17.3	7.3	
Visit 5 (*n* = 95)			
ECOG 0–1	28.0	7.5	0.004
ECOG > 1	18.9	10.6	
Visit 6 (*n* = 76)			
ECOG 0–1	27.8	7.3	0.036
ECOG > 1	19.0	8.9	
Visit 7 (N = 76)			
ECOG 0–1	27.3	7.6	0.005
ECOG > 1	17.3	8.1	

*ECOG PS* Eastern Cooperative Oncology Group Performance Status

*SD* Standard Deviation

We also evaluated the values of BOMET-QoL-10 scores for those patients (up to 42 patients between visits 4 and 5) reporting their perceived health status between visits remaining “more or less the same.” As expected, their corresponding BOMET-QoL-10 scores did not vary between consecutive visits either. There were 26 highly stable patients who reported the same perceived health status from baseline all throughout the last follow-up. As expected, BOMET-QoL-10 global mean for these highly stable patients was unchanged between baseline and visit 7 (26.4, SD = 8.8 and 26.4, SD = 7.0, respectively) (*p* = 0.956). Thus, BOMET-QoL-10 scores for highly stable patients were consistently stable throughout the study.

**Fig. 2:**
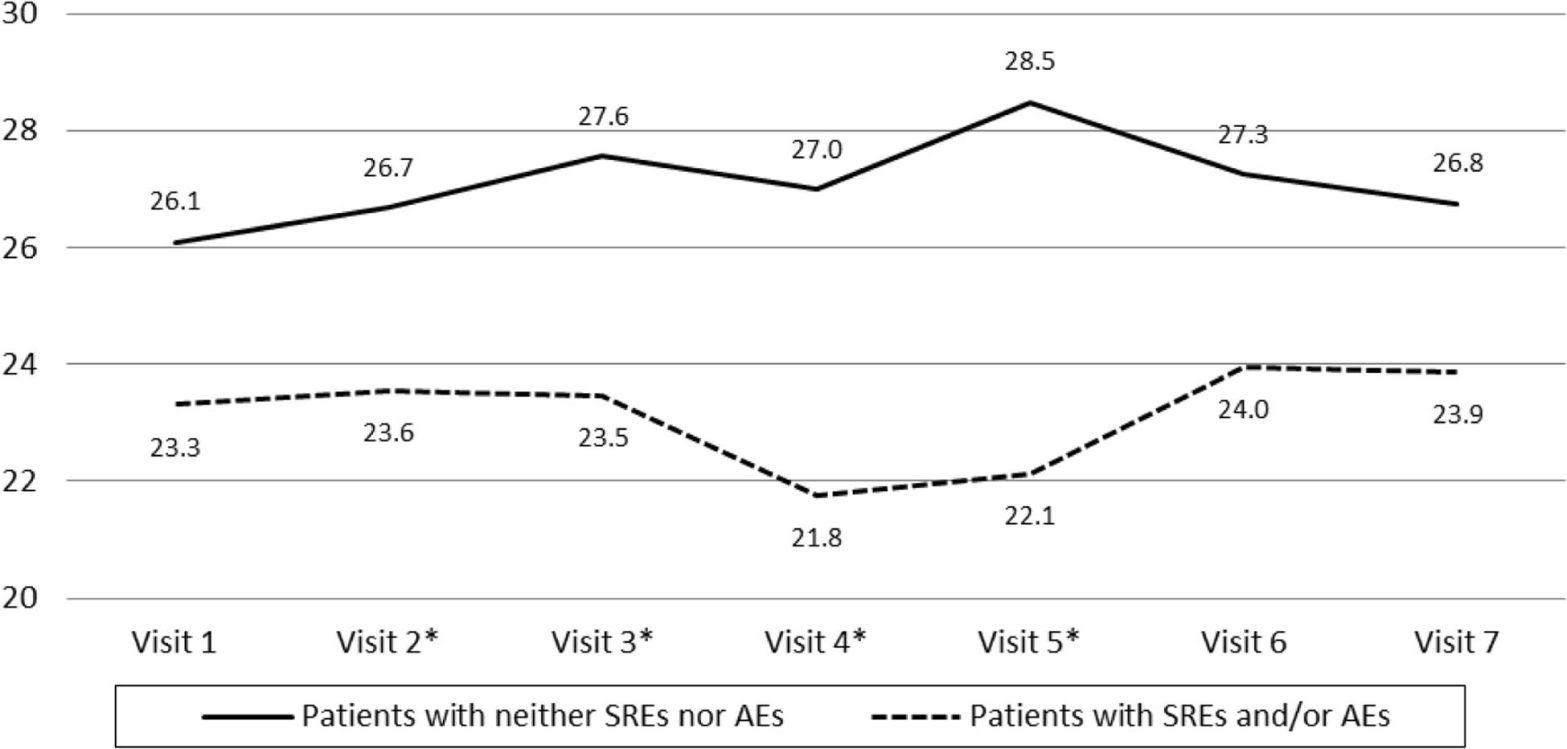
BOMET-QoL-10 mean scores for each visit over time reported by patients with AEs and/or SREs versus patients with neither. Higher scores indicate better Health Related Quality of Life.*Mean scores are statistically different between patient groups (*p* < 0.05)

In addition we compare the BOMET-QoL-10 scores between patients with SREs *and/or* AEs and patients with neither, as well as the questionnaire’s power of discrimination between these two groups, we compared their average scores for each visit (Fig. 2). Results show that the average score for each group remained fairly stable (range: 21.8 to 24.0 for the former and range: 26.1 to 28.5 for the latter). The mean differences between the two groups were in the expected direction and statistically significant in 4 of the 7 visits while approaching significance in the other 3 visits. Further, we tested the difference between the overall average score (all 7 visits) between patients ever reporting SREs and/or AEs at any of the visits during the 2 year-follow up (*n* = 111; mean = 24.1, SD = 7.2) and that for patients reporting neither SREs nor AEs at any of the visits during the same period (*n* = 61; mean = 26.8, SD = 7.4). The average score for the first group was significantly lower than that of the second group (*p* ≤ 0.007) (data not shown).

Finally, MBC patients receiving **specific treatment** for BM reported a clinically significant improvement in HRQoL throughout the study compared to those patients who did not. The magnitude of the treatment’s effect for BM was calculated as the difference between the BOMET-QoL-10 mean values at visit 7 in these two groups divided by a combined standard deviation. The relative overall study variability yielded an effect size of 0.203 in BOMET-QoL-10 scores, which is greater than our pre-set value for clinical significance (0.200) (Table 4)).

**Table 4:** Effect size of the global BOMET-QoL-10 mean values at visit 7 in patients receiving specific treatment for BM vs those who did not

BM intervention	Visit 7				
	Mean	SD	N = 83	%	D
No	27.08	7.24	24	28.9%	0.203
Yes	25.41	8.61	59	71.1%	

Abbreviations: *D* Effect size

## Discussion

With increasing rates of survival after a breast cancer diagnosis a growing number of patients are at risk for MBC [1, 19]. For MBC patients, whose treatments are essentially palliative, an accurate and clinically valid assessment of HRQoL is particularly important. Thus, instruments should be comprehensive enough to capture the key aspects of a patients´ quality of life, though still easy to understand and brief. This could remain as a feasible tool to be used in clinical practice [20, 21].

Aside from BOMET-QoL, QLQ-BM22, a widely used tool, is the only other HRQoL instrument designed specifically for BM patients. QLQ-BM22 is a useful and reliable instrument especially when administered in combination with the QLQ-C30 [22, 23]. Several studies have evaluated and validated the QLQ-BM22 module with male and female patients of a variety of primary cancers and BM, and/or residing in a variety of countries as summarized below. However, despite its satisfactory psychometric properties, caution is warranted when comparing certain items by gender or country as they may function differently [24]. Raman and colleagues (2016) [23] validated the module based on a sample of 204 men and women diagnosed with one of at least six different cancers and BM. Patients were assessed at baseline and at a 42 day-follow-up. Another international study evaluated the module’s performance based on two administrations—1 month apart— to 59 patients from 6 different countries [9]. A larger 7-country study administered the module twice—1 month apart—to 400 male and female patients diagnosed with at least 9 different cancers with BM [25]. Recently, Lin and Pakpour (2016) [24] performed a psychometric evaluation of QLQ-BM22 based on a pre-treatment single administration of a pooled sample of 573 BM patients from 8 countries. Miki-Rosário and colleagues (2016), [26] validated the Brazilian Portuguese translation of the QLQ-BM22 based on a sample of 95 men and women with BM derived from 9 different cancers. Reliability, face and content validity were assessed based on 40 patients over three administrations of the module. The use of other tools not designed specifically for BM patients (e.g., Brief Pain Inventory, the QLQ-C30 alone, the QLQ-C15 PAL, the Net pain relief concept, and the Spitzer’s QOL Index) to assess QOL in patients with BM has been reviewed by McDonald and colleagues [8].

BOMET-QoL-10 has been shown to closely reflect variations in relevant pain and wellness measures, having good internal consistency, and being highly reliable among cancer patients with BM [18, 22]. Our objective in this study was to evaluate whether the BOMET-QoL-would be a valuable tool to use for breast cancer patients with BM. The questionnaire was tested at baseline on a MBC patient population in which 70% of individuals reported symptoms, close to a quarter experienced irruptive pain, over half presented chronic conditions, and more than half were receiving chemotherapy. BOMET-QoL-10 proved to be a quick assessment tool performing well in terms of readability and completion time (about 10 min); and presenting very low levels of missing or invalid data (0–1.2%) throughout all 7 follow-up visits. Quick completion is important in the context of clinical practice not only because of the inherent time limitations but also because it reduces response burden in patients who may be experiencing chronic pain and/or who may fatigue easily during assessments. Thus, single instruments are preferred over two or more [27]. Whereas succinctness is important, items highly relevant to the patient’s experience are also key to keeping subject burden low [21]. Our results convey low administration burden and high content relevancy. Acceptable subject burden is a key factor for achieving high completion and response rates as well as high data quality [28].

BOMET-QoL-10 scores for overall HRQoL remained stable over time for the sample as a whole. This lack of variability may reflect the influence of aspects other than the socio-demographic and clinical factors assessed here. However, when patients were grouped by disease status (e.g., with or without SRE and/or AE) BOMET-QoL-10 scores reflected the differences in group wellness one would expect at each visit and throughout the study (had SREs and/or AEs at *any* of the visits vs. not). Further, the instrument’s scores were significantly correlated with physician-reported patient status, patient-reported pain, symptoms, and perceived health status. Perceived pain was directly proportional to HRQoL and VAS scores and BOMET-QoL-10 scores also varied accordingly as patient’s pain improved or worsened. As expected, the BOMET-QoL-10 and ECOG PS scores were inversely proportional throughout the study. This supports previously reported results based onQLQ-C30 and QLQ-BM22 [23, 25, 29]. We should highlight that in some of the categories, the effect was the opposite, but this was most likely due to the low number of patients in those categories. Overall, these results suggest that the BOMET-QoL-10 is a reliable and useful, tool to measure of HRQoL in this patient population.

Finally, we assessed the instrument’s sensitivity to change by calculating the effect size for patients who had received BM-specific medical intervention versus those who had not. We then compared it to an a priori value we considered to be minimum clinically relevant effect. The BOMET-QoL-10 effect size was greater than the pre-set value. Thus, MBC patients receiving specific treatment for BM reported a clinically relevant improvement in HRQoL throughout the study compared to MBC patients who did not. This confirms that the instrument is sensitive to changes independently of the statistical significance of other results [16].

The study presents limitations which should be considered when interpreting our results. First, a majority of patients (71.5%) were recruited by 5 of the 15 participant research centers. As palliative care populations tend to be highly heterogeneous, a limited number of recruiting centers may have resulted in a less diverse sample of participants than expected. This may have introduced an unknown bias [30] and reduced the generalizability of the results. However, it is not uncommon for samples to be drawn from one or a handful of medical facilities [29, 31]. Second, due to feasibility concerns the study is based on a non-probability sample which removes the possibility of estimating sampling variability or to ascertain potential biases. Research centers such as Statistics Canada, nonetheless, use non-probability sampling for questionnaire testing [32].

A third limitation is the small number of patients completing the entire 24-month study. This is the result of an initial small sample size compounded by a much higher lost-to-follow-up rate (51.7%) than anticipated (20%). However, recent adaptation and validation studies of HRQoL instruments as well as HRQoL assessments of palliative care patients have been based on small samples followed for short periods of time ranging from 1 to 6 months [9, 23, 26].

Unfortunately, the lower numbers limited the evaluation of the study’s secondary objectives and actual differences may have gone undetected given the low statistical power of some of our comparisons. For instance, even though BM-associated pain is quite common and often undertreated in MBC patients [33], BOMET-QoL-10 failed to statistically differentiate the HRQoL of patients with AEs and/or SREs versus those with neither one in 3 of the 7 visits even when they were substantial in magnitude and clearly of clinical significance. These differences, which approached significance, most likely would have reached significance in a larger sample. In fact, when we compared patients ever reporting SREs and/or AEs during the 2-year study (*n* = 111) to those who did not report either during the study (*n* = 61) their overall average scores were significantly different (*p* < 0.007).

The study also has some important strengths. First, the longitudinal administration of the same tool to patients with BM in seven occasions over a 2-year period represents a solid assessment of the user-acceptance, respondent burden of the tool, and stability of the measurement. As aforementioned, this contrasts with other studies which include just one or two follow-ups over periods of time under 1 year [9, 16, 18, 23, 26], with the exception of Jordhøy and colleagues who followed 395 patients for 2 years [29]. Second, our main overall outcome, patients´ HRQoL, was assessed by three methods in addition to BOMET-QoL-10: the doctor’s perception, the patient’s perception, and the VAS score. These assessments were highly associated suggesting that BOMET-QoL-10 has a high construct validity. Third, certain dimensions of HRQoL are subjective and may be affected by gender, site of original tumor [16], type of therapy administered or cultural norms and expectations in unknown ways. Our results minimize this potential negative interaction by using a fairly homogenous patient sample (female MBC patients residing in Spain). In contrast, many of the studies evaluating or using QLQ-BM22 or BOMET-QoL-10 are based on patients of both genders, diagnosed with different primary cancers, and/or recruited across countries with different cultures and languages [9, 16, 18, 23, 26, 29].

## Conclusions

The regular surveillance of HRQoL issues in patients with advanced disease and receiving palliative care can be used to guide pain management therapy [34] and other symptoms and choose the best course of palliative action for patients [26]. The development of quality easy-to-administer tools such as BOMET-QoL-10 will facilitate such needed surveillance. Although, not as comprehensive as other assessments, BOMET-QoL-10’s brevity and relevancy allow the clinician to accurately and efficiently assess some relevant quality of life aspects in patients with breast cancer and BM. In only 10 min and without burdening symptomatic frail patients, this reliable and useful tool identifies key quality of life aspects in a regular clinical setting.

This instrument exhibits longitudinal and cross-validation, construct validity, as well as feasibility and it is sensible to detect some changes related with the disease evolution and their treatment. BOMET-QoL-10 is sensitive to the number of symptoms, pain level, ECOG PS, presence of SREs and/or AEs, and the effects of BM-specific treatment.

Further research is needed to test this tool in larger samples of breast cancer patients with BM as well as in other homogenous cancer populations with BM, e.g., prostate or lung cancer patients with BM. Further steps would involve translation and cross-cultural adaptation of the tool to other languages and countries.

## Supplementary information

**Additional file 1.** BOMET-QoL 10 points items

**Additional file 2.** Breast Cancer Treatments at baseline

**Additional file 3.** Treatments for bone metastasis per visit

**Additional file 4.** BOMETQoL items evolution through study visits and mean standardized global score

**Additional file 5.** Correlation between the number of symptoms at visit 7 and the BOMET-QoL global score

## Data Availability

The datasets used and/or analyzed during the current study are available from the corresponding author on reasonable request.

## Abbreviations

AE: Adverse Events
BM: Bone Metastasis
BOMET: Bone Metastasis Quality of Life
ECOG PS: Eastern Cooperative Oncology Group Performance Status
EORTC: European Organization for Research and Treatment of Cancer
FACT: Functional Assessment of Cancer Therapy
HRQoL: Health Related Quality of Life
MBC: Metastatic Breast Cancer
PMI: Pain Management Index
SD: Standard Deviation
SREs: Skeletal-Related Events
VAS: Visual Analog Scale
