# Supplementary material for: BOMET-QoL-10 questionnaire for breast cancer patients with bone metastasis: the prospective MABOMET GEICAM study
Source: J Patient Rep Outcomes. 2019 Dec 21;3:72. doi: 10.1186/s41687-019-0161-y (PMC6925605; doi:10.1186/s41687-019-0161-y)
Supplement: Supplementary file 1 — Additional file 1. BOMET-QoL 10 points items [file 41687_2019_161_MOESM1_ESM.docx]

**Items:**

- I feel tired
- I find it difficult to get up
- I have general malaise
- I feel depressed and want to cry
- I do not want to go out much
- I avoid activities with my family
- I have pain in some parts of my body, such as my back and legs, that affects my life
- I have permanent pain that is affecting my life
- I have intense pain that never eases
- The pain prevents me from enjoying life as I used to

Answer score:

- Always (0), Nearly always (1), Sometimes (2), Rarely (3), Never (4).
