# Supplementary material for: BOMET-QoL-10 questionnaire for breast cancer patients with bone metastasis: the prospective MABOMET GEICAM study
Source: J Patient Rep Outcomes. 2019 Dec 21;3:72. doi: 10.1186/s41687-019-0161-y (PMC6925605; doi:10.1186/s41687-019-0161-y)
Supplement: Supplementary file 2 — Additional file 2. Breast Cancer Treatments at baseline [file 41687_2019_161_MOESM2_ESM.docx]

| Breast Cancer Treatments at baseline | **N** | **%** |
| --- | --- | --- |
|  | **N** | **%** |
| **Chemotherapy** | 94 | 54,7 |
| 1^st^ line | 55 | 58,5 |
| 2^nd^ line | 30 | 31,9 |
| 3^rd^ line | 12 | 12,8 |
| **Radiotherapy** | 10 | 5,8 |
| **Surgery** | 7 | 4,1 |
| **Hormonotherapy** | 100 | 58,1 |
| 1st line | 59 | 59,0 |
| 2^nd^ line | 38 | 38,0 |
| 3^rd^ line | 5 | 5,0 |
| **Other** | 21 | 12,2 |

One patient could be taken more than one treatment
