# Supplementary material for: BOMET-QoL-10 questionnaire for breast cancer patients with bone metastasis: the prospective MABOMET GEICAM study
Source: J Patient Rep Outcomes. 2019 Dec 21;3:72. doi: 10.1186/s41687-019-0161-y (PMC6925605; doi:10.1186/s41687-019-0161-y)
Supplement: Supplementary file 3 — Additional file 3. Treatments for bone metastasis per visit [file 41687_2019_161_MOESM3_ESM.docx]

| **Treatments for bone metastasis** | | | | | | | | | | | | | | |
| --- | --- | --- | --- | --- | --- | --- | --- | --- | --- | --- | --- | --- | --- | --- |
|  | **visit 1** | | **visit 2** | | **visit 3** | | **visit 4** | | **visit 5** | | **visit 6** | | **visit 7** | |
|  | **n=172** | | **n=152** | | **n=134** | | **n=124** | | **n=106** | | **n=84** | | **n=83** | |
|  | **n** | **%** | **n** | **%** | **n** | **%** | **n** | **%** | **n** | **%** | **n** | **%** | **n** | **%** |
| **Palliative radiotherapy** | 10 | 5,8 | 11 | 7,2 | 10 | 7,5 | 8 | 6,5 | 2 | 1,9 | 2 | 2,4 | 0 | 0,0 |
| **Chemotherapy** | 22 | 12,8 | 1 | 0,7 | 9 | 6,7 | 4 | 3,2 | 1 | 0,9 | 1 | 1,2 | 1 | 1,2 |
| **Surgery for bone metastasis** | 0 | 0,0 | 2 | 1,3 | 0 | 0,0 | 0 | 0,0 | 0 | 0,0 | 0 | 0,0 | 0 | 0,0 |
| **Analgesics** | 65 | 37,8 | 17 | 11,2 | 19 | 14,2 | 16 | 12,9 | 6 | 5,7 | 6 | 7,1 | 5 | 6,0 |
| **Biphosphonates** | 136 | 79,1 | 29 | 19,1 | 25 | 18,7 | 22 | 17,7 | 18 | 17,0 | 18 | 21,4 | 11 | 13,3 |
| Zoledronato | 133 | 97,8 | 29 | 100,0 | 25 | 100,0 | 21 | 95,5 | 18 | 100,0 | 18 | 100,0 | 10 | 90,9 |
| Pamidronato | 2 | 1,5 | 0 | 0,0 | 0 | 0,0 | 0 | 0,0 | 0 | 0,0 | 0 | 0,0 | 0 | 0,0 |
| Ibandronato | 1 | 0,7 | 0 | 0,0 | 0 | 0,0 | 0 | 0,0 | 0 | 0,0 | 0 | 0,0 | 0 | 0,0 |
| Otros **^Especificar^** | 0 | 0,0 | 0 | 0,0 | 0 | 0,0 | 1 | 4,5 | 0 | 0,0 | 0 | 0,0 | 1 | 9,1 |
