# Supplementary material for: BOMET-QoL-10 questionnaire for breast cancer patients with bone metastasis: the prospective MABOMET GEICAM study
Source: J Patient Rep Outcomes. 2019 Dec 21;3:72. doi: 10.1186/s41687-019-0161-y (PMC6925605; doi:10.1186/s41687-019-0161-y)
Supplement: Supplementary file 4 — Additional file 4. BOMETQoL items evolution through study visits and mean standardized global score [file 41687_2019_161_MOESM4_ESM.docx]

|  | **visit 1** | | | **visit 2** | | | **visit 3** | | | **visit 4** | | | **visit 5** | | | **visit 6** | | | **visit 7** | | |  |
| --- | --- | --- | --- | --- | --- | --- | --- | --- | --- | --- | --- | --- | --- | --- | --- | --- | --- | --- | --- | --- | --- | --- |
|  | **n=172** | | | **n=152** | | | **n=134** | | | **n=124** | | | **n=106** | | | **n=84** | | | **n=83** | | | **p** |
| **Bomet-QoL Questionnaire** | **N** | **Mean** | **SD** | **N** | **Mean** | **SD** | **N** | **Mean** | **SD** | **N** | **Mean** | **SD** | **N** | **Mean** | **SD** | **N** | **Mean** | **SD** | **N** | **Mean** | **SD** |  |
| 1. I feel tired | 171 | 1,9 | 0,9 | 152 | 1,9 | 0,9 | 133 | 1,9 | 0,9 | 124 | 1,9 | 0,9 | 105 | 2,0 | 1,0 | 84 | 2,1 | 0,9 | 83 | 1,9 | 0,9 | 0,79 |
| 2. I find it difficult to get up | 171 | 2,4 | 1,3 | 152 | 2,5 | 1,2 | 133 | 2,5 | 1,1 | 123 | 2,4 | 1,2 | 105 | 2,6 | 1,2 | 84 | 2,5 | 1,2 | 83 | 2,5 | 1,3 | 1,00 |
| 3. I have general malaise | 171 | 2,4 | 1,1 | 152 | 2,4 | 1,1 | 134 | 2,6 | 1,0 | 124 | 2,4 | 1,1 | 105 | 2,4 | 1,0 | 84 | 2,5 | 1,0 | 82 | 2,5 | 1,1 | 0,37 |
| 4. I feel depressed and want to cry r | 170 | 2,8 | 1,0 | 152 | 2,8 | 1,0 | 134 | 2,9 | 1,0 | 123 | 2,8 | 1,0 | 105 | 2,9 | 1,1 | 84 | 2,9 | 1,0 | 83 | 2,9 | 1,0 | 0,80 |
| 5. I do not want to go out much | 171 | 2,5 | 1,2 | 151 | 2,5 | 1,1 | 134 | 2,6 | 1,1 | 124 | 2,5 | 1,2 | 105 | 2,6 | 1,2 | 84 | 2,7 | 1,1 | 83 | 2,6 | 1,1 | 0,74 |
| 6. I avoid activities with my family | 171 | 3,2 | 1,0 | 151 | 3,2 | 0,9 | 134 | 3,2 | 1,0 | 123 | 3,1 | 1,0 | 106 | 3,1 | 1,0 | 84 | 3,3 | 0,9 | 83 | 3,1 | 1,1 | 0,08 |
| 7. I have pain in some parts of my body, such as my back and legs, that affects my life | 171 | 2,0 | 1,1 | 152 | 2,0 | 1,1 | 134 | 2,2 | 1,2 | 123 | 2,0 | 1,1 | 106 | 2,1 | 1,2 | 84 | 2,0 | 1,1 | 83 | 2,0 | 1,1 | 0,21 |
| 8. I have permanent pain that is affecting my life | 171 | 2,8 | 1,3 | 150 | 2,7 | 1,3 | 134 | 2,7 | 1,3 | 122 | 2,7 | 1,2 | 106 | 2,8 | 1,2 | 84 | 2,8 | 1,2 | 83 | 2,7 | 1,2 | 0,11 |
| 9. I have intense pain that never eases | 171 | 3,2 | 1,0 | 152 | 3,2 | 1,0 | 133 | 3,2 | 1,0 | 123 | 3,1 | 1,0 | 106 | 3,2 | 1,1 | 84 | 3,0 | 1,1 | 82 | 3,0 | 1,1 | 0,01 |
| 10. The pain prevents me from enjoying life as I used to | 171 | 2,6 | 1,3 | 152 | 2,6 | 1,2 | 134 | 2,6 | 1,3 | 123 | 2,6 | 1,2 | 106 | 2,7 | 1,2 | 84 | 2,7 | 1,2 | 83 | 2,6 | 1,1 | 0,19 |
| **Questionnaire mean standarized global score** | **170** | **64,3** | **1,5** | **149** | **64,6** | **1,6** | **131** | **65,9** | **1,6** | **122** | **64,4** | **1,9** | **105** | **66,2** | **2,1** | **84** | **66,1** | **2,2** | **82** | **64,7** | **2,3** | **0,15** |

Score: 0 - Always , 1- Nearly always, 2 - Sometimes, 3 - Rarely, 4 - Never

Questionnaire mean standarized global score: min 0 - max 100

p. statistical signification between visit 1 and 7

Missings: We consider missing values those questionnaires where one question is not answered

Visit 1= 2 missings

Visit 2= 3 missings

Visit 3= 3 missings

Visit 4= 2 missings

Visit 5= 1 missings

Visit 6= 0 missings

Visit 7= 1 missings
