# Supplementary material for: BOMET-QoL-10 questionnaire for breast cancer patients with bone metastasis: the prospective MABOMET GEICAM study
Source: J Patient Rep Outcomes. 2019 Dec 21;3:72. doi: 10.1186/s41687-019-0161-y (PMC6925605; doi:10.1186/s41687-019-0161-y)
Supplement: Supplementary file 5 — Additional file 5. Correlation between the number of symptoms at visit 7 and the BOMET-QoL global score [file 41687_2019_161_MOESM5_ESM.docx]

|  | **Mean global BOMET-QoL score at v7** | | | | **Correlation** | **p** |
| --- | --- | --- | --- | --- | --- | --- |
|  | **Mean** | **SD** | **n** | **%** |  |  |
| No symptoms | 31,08 | 6,37 | 13 | 15,7% | -0,41 | 0,000 |
| 1 symptom | 32,00 | 6,59 | 8 | 9,6% |  |  |
| 2 symptoms | 26,64 | 8,08 | 11 | 13,3% |  |  |
| 3 symptoms | 24,88 | 8,56 | 8 | 9,6% |  |  |
| 4 symptoms | 26,80 | 5,67 | 5 | 6,0% |  |  |
| 5 symptoms | 26,50 | 7,12 | 6 | 7,2% |  |  |
| 6 symptoms | 24,25 | 9,54 | 4 | 4,8% |  |  |
| 7 symptoms | 21,89 | 9,69 | 9 | 10,8% |  |  |
| 8 symptoms | 19,00 | 6,98 | 4 | 4,8% |  |  |
| 9 symptoms | 16,33 | 4,04 | 3 | 3,6% |  |  |
| 10 symptoms | 23,00 | 8,49 | 2 | 2,4% |  |  |
| 11 symptoms | 22,00 | 7,87 | 4 | 4,8% |  |  |
| 12 symptoms | 25,40 | 9,86 | 5 | 6,0% |  |  |
| 13 symptoms | 24,00 | . | 1 | 1,2% |  |  |
